# Oro-Dental Health of Patients with Chronic Hepatic Failure

**Published:** 2020

**Authors:** M. Zahed, M. Bahador, M. K. Hosseini Asl, F. Lavaee, A. Azad, A. Bahador

**Affiliations:** 1 *Oral and Dental Disease Research Center, Department of Oral and Maxillofacial Medicine, School of Dentistry, Shiraz University of Medical Sciences, Shiraz, Iran*; 2 *Shiraz Transplant Center, Namazee Hospital, Shiraz University of Medical Sciences, Shiraz, Iran*

**Keywords:** Chronic liver failure, Oral mucosa, Xerostomia, Oral health

## Abstract

**Background:**

Patients with chronic liver failure (CLF) faced serious medical conditions including the oral cavity.

**Objective:**

To investigate the prevalence of oral mucosal lesions, saliva flow rate, and dental complications in candidates of liver transplant surgery.

**Methods:**

In this cross-sectional study, oral and dental health of 77 patients with CLF and 77 healthy individuals were assessed for oral mucosal lesions, salivation rate, DMFT (decayed, missing, filled teeth) index, and bone level. To carefully determine the indices and examine the patients thoroughly, a panoramic radiography was also taken from each participant.

**Results:**

The frequency of oral mucosal lesions in patients was significantly (p<0.001) higher than the comparison group. The most frequent lesion identified was angular cheilitis followed by candidiasis. The mean saliva flow rate in the patients (0.85 g/min) was also significantly (p<0.001) lesser than that in healthy individuals (1.58 g/min). The DMFT index and bone level were not significantly different between the two groups. Nor was a correlation between the MELD score and each of DMFT index, bone loss, or oral mucosal lesions.

**Conclusion:**

Mucosal lesions, especially fungal-related lesions, are more prevalent in the oral cavity of patients with CLF. The saliva production rate is reduced due to various medications used in this group. Patients with CLF are prone to oral infections and a thorough oro-dental examination is crucial in this group of patients. Vigorous oral hygiene instructions should be offered to liver cirrhosis individuals.

## INTRODUCTION

End-stage liver disease or cirrhosis is a consequence of long-term damage to the liver tissues [1]. Hepatic impairment affects all body systems including the oral cavity. Cholestatic liver disease affects bone structure and can seriously affect teeth and jaws [2]. The only treatment available for end-stage liver disease is liver transplantation. Although this is crucial for the patient, special care and watchful precautions are also needed for many years after transplantation [3].

Oro-dental complications, as a common source of infection, should be monitored and treated before performing transplantation surgeries. An oral examination and an approval from a dentist implying absence of any signs of infection are required [4]. This protocol is performed because immunosuppressive drugs such as tacrolimus and cyclosporine, which are prescribed after transplantation, may predispose the recipient to sepsis and infection [5]. Elimination of any infections and their foci is therefore recommended in almost all patients referred for liver transplantation [6].

There is a lack of evidence about the advantage of an optimal dental examination prior to transplantation [7]. In addition, there is no documented study examining if oral manifestations and mucosal lesions are related to end-stage liver disease [8]. 

There are reports on oral mucosal manifestations after liver transplantation. Lesions such as fissured tongue, candidiasis, increased risk of viral infections such as herpes simplex virus type 1 and 2 or cytomegalovirus, graft versus host disease and oral cancers are more prevalent post-transplantation [9]. Furthermore, patients who undergo liver transplant surgery, often take several medications with different side effects that affect oro-dental conditions including xerostomia and hyposalivation, which could lead to increased risk of oral infection and subjective dry mouth syndrome. This hyposalivation is augmented when the daily dose and the number of drugs are increased [10]. Moreover, a careful examination of the lips and mucosal tissues of the oral cavity for detecting possible neoplastic lesions, is crucial after transplantation [11].

We conducted this study to investigate different oral mucosal lesions and dental complications in candidates of liver transplantation as compared with a group of normal individuals. 

## PATIENTS AND METHODS

Patients

Liver transplantation in Iran is centralized in Nemazee Hospital, Shiraz, southern Iran. This cross-sectional study was thus conducted on patients who were referred to Imam Reza Dental Clinic affiliated to Shiraz University of Medical Sciences. All adult patients above 18 years who were referred to the center in summer and autumn of 2018 were enrolled in this study. The inclusion criteria were those with the initial diagnosis of chronic liver failure who were referred to an oral and maxillofacial medicine specialist for dental health approval prior to transplantation. The main etiologies of liver disease were primary sclerosing cholangitis (PSC), autoimmune hepatitis (AIH), hepatitis B or C, non-alcoholic steatohepatitis (NASH), cryptogenic hepatitis, and liver tumors. This had been confirmed by pathologic evaluation and clinical examinations by the transplantation team members. The exclusion criteria included patients who had no pathologic confirmation, incomplete documents or missing laboratory tests. Patients who were not willing to participate were also excluded from the study.

The patients were categorized according to their MELD score into three groups—those with a low score (MELD≤10), medium (MELD: 11–18), and high (MELD≥19).

The patients’ medications and other systemic diseases they had (e.g., diabetes and cardiovascular diseases) were recorded as well. Serum ALT and AST levels, platelet count, INR, MELD score, blood type, and the etiology of the liver disease were also recorded from the patients’ latest laboratory tests. Information regarding gastrointestinal complaints, skin problems (e.g., pruritus and rashes), ascites, and jaundice were gathered from a questionnaire or a direct interview with patients and their family members.

Patients’ medications were recorded and categorized into six groups—antihypertensive drugs, anti-inflammatory drugs, tranquilizers, gastrointestinal drugs, cardiovascular drugs, and immunosuppressive agents.

Comparison group

The comparison group was selected from family members or other patients who attended Shiraz Dental School Clinic during the same period with no oral or dental problems. Those who had a systemic disease or used any medications with oral manifestations were also excluded from the study. The age and sex of the comparison group was matched with the patients. 

Imaging Procedures 

Panoramic views were prepared by a Planmeca XC Proline panoramic machine (Helsinki, Finland). Exposure factors were adjusted according to the size and age of the patients (57–85 kVp, 10 mA), using Agfa PSP receptors (Germany). The images were observed on a Barco monitor (China) in a semi-darkened room. All radiographs were evaluated by two oral and maxillofacial medicine specialists to achieve a capital value of agreement. The film was examined to measure the DMFT index, evaluation of bone levels and detection of mandibular lesions. Bone loss was evaluated according to the level of bone from the CEJ of existing teeth on radiography. 

Oral Examination

The oral cavity and dentition examinations of the patients and the comparison group members were performed by a dentist who was trained by two oral and maxillofacial medicine and hospital dentistry specialists. Every examination included a careful clinical visual and tactile inspection of the mucosal and gingival tissues of the oral cavity including the lips, hard and soft palates, floor of the mouth, inner cheeks, and tongue and vermilion border for any possible mucosal and gingival lesions or pathology. Ulcers and erosions, exophytic lesions, hyperkeratosis and erythematous mucosa, pigmentations, and other abnormal variations of the oral mucosa were recorded. If indicated, biopsy and histopathological evaluation, cytology, and culture were performed to reach a definitive diagnosis of mucosal lesions. In this examination, normal variation such as fissured tongue, geographic tongue, and physiologic pigmentation were excluded. The teeth were also thoroughly examined by visual and tactile examination using an explorer to assess the DMFT index according to the recommended protocols for oral health surveys [12]. The same examinations were performed for the comparison group.

Saliva Flow Rate

To evaluate unstimulated saliva hypofunction, the patients were asked to refrain from intake of any food or beverage one hour before the test session. Smoking, chewing gum and intake of coffee were also prohibited during this hour. The subjects were advised to rinse their mouth several times with deionized distilled water and then to collect their saliva in a utensil in one minute and then the weight of the saliva was recorded. The salivary flow rate was calculated according to the following equation:

An “unstimulated hypofunction” was considered when the value was <0.1 g/min [13].

Ethical Considerations

An informed written consent was obtained from all participants at the time of enrollment. The study protocol was approved by the Ethics Committee of Shiraz University of Medical Sciences. 

Statistical Analysis

Data were analyzed with SPSS^®^ for Windows^®^ ver 22. χ^2^ test was used to compare qualitative variables between the patients and the comparison group. Pearson’s correlation coefficient was used to assess the association between MELD score and other indices measured. Student’s t test for independent samples was employed to compare mean MELD score between the two groups. A p value <0.05 was considered statistically significant.

## RESULTS

Ninety-one patients were studied; 14 were excluded because their documents and laboratory tests were incomplete, leaving 77 (54 male) patients for analyses. The patients had a mean age of 42.6 (range 19–60) years. The comparison group included 49 (64%) male and had a mean age of 43.2 years. 

Thirty-one (40%) patients complained of gastrointestinal problems; 43% had pruritus and 27% complained of skin problems such as rashes and acne. Fifty-four percent of the studied patients were using gastrointestinal drugs; 12%, antihypertensive drugs; 31%, cardiovascular drugs; 9%, tranquilizers; 14%, immunosuppressive agents; and 17%, anti-inflammatory drugs.

The most frequent indication for liver transplantation was PSC (20%) followed by AIH (16%), hepatitis B (13%), cryptogenic hepatitis (12%), NASH (12%), Buddchiari disease (10%), liver tumors (9%), hepatitis C (4%), hydatid cyst (3%), and alcoholic cirrhosis (3%). No association was found between the MELD score and the etiology of liver failure.

**Figure 1:**
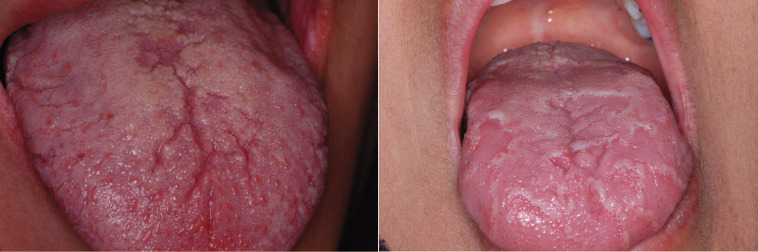
Fissured tongue and geographic tongue in patients with liver cirrhosis

**Figure 2:**
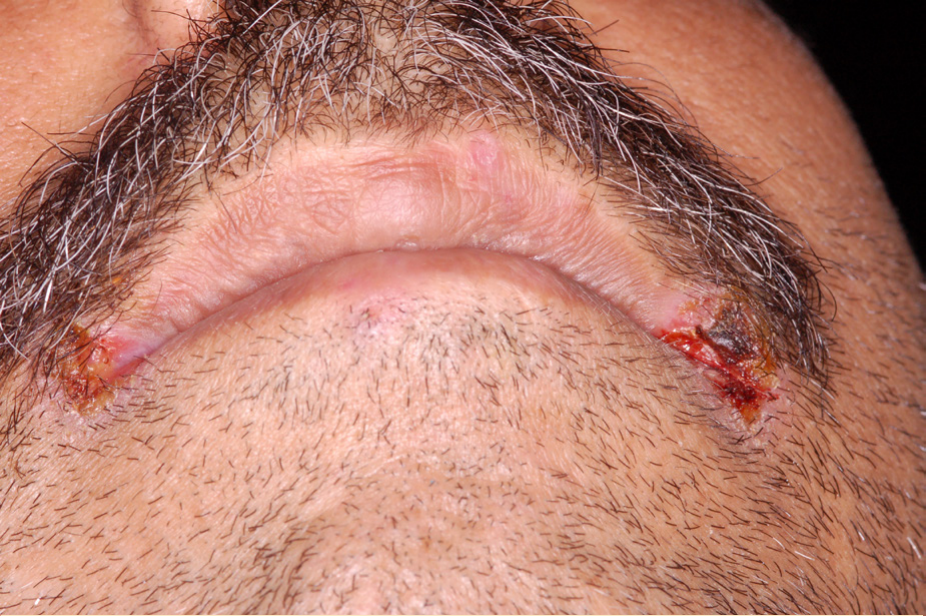
Angular cheilitis in a patient with liver failure

**Figure 3:**
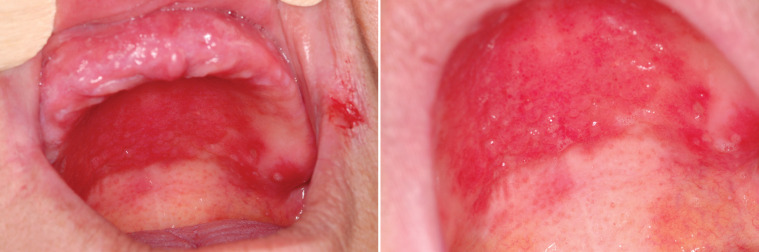
Denture stomatitis (candidiasis) in a patient with liver failure

If fissured tongue and geographic tongue were taken as normal variants of the oral mucosa, patients with end-stage liver disease were more likely to develop mucosal lesions compared with normal individuals (OR=11.4, 95% CI: 4.1–31.3) (Fig 1). The most frequent lesion in the patients was angular cheilitis followed by candidiasis (Table 1, Figs 2 and 3).

**Table 1:** Frequency of oral mucosal lesions in liver cirrhosis patients and normal individuals

Mucosal lesions	Group		Total
	Patients	Control	
Angular cheilitis	13	1	14
Hyperkeratosis	2	1	3
Candidiasis	9	0	9
Epulis fissuratum	3	1	4
Traumatic ulcers	6	2	8
Lichen planus	1	0	1
None	43	72	115
Total	77	77	154

The mean saliva weight was significantly (p<0.001) lower in patients (0.85 g/min) compared with the comparison group (1.58 g/min). The mean DMFT score was 15.2 in the patients and 14.6 in the comparison group (p=0.618). Neither the MELD score nor the etiology of disease had significant association with DMFT index.

Fort-nine patients had bone loss detected in their panoramic radiography. The prevalence of bone loss was not significantly different between the patients and the comparison group. The MELD score had no significant effect on the level of bone loss.

## DISCUSSION

There are just few studies on oral mucosal health in patients with liver cirrhosis and candidates of liver transplantation [14]. We found that oral mucosal lesions are significantly more prevalent among those with chronic liver failure as compared with normal individuals. Moreover, saliva secretion was lesser in this group compared to normal individuals.

Angular cheilitis was the most common lesion identified in patients with liver cirrhosis. It was significantly more common in those with chronic hepatic failure as compared with healthy individuals. Angular cheilitis that manifests as ulcer, erythema, and crusting at the corners of the mouth, results from microbial growth, mainly Candida albicans, due to immunosuppressive states, vertical dimension changes, iron and vitamin deficiencies, and gastrointestinal disorders [15, 16]. Other forms of candidiasis such as denture stomatitis and erythematous candidiasis were also common in the studied patients studied. Fifty percent of our study group were receiving immunosuppressive agents; 17% were using anti inflammatory drugs. These drugs place the patient in an immunosuppressive state, which augments the growth of opportunistic infections such as Candida albicans. Candidial infection was also reported in other studies of patients with liver cirrhosis [6, 14]. The use of diuretics, smoking, and diabetes are mentioned as the cause for this mucosal lesion in patients with chronic hepatic failure [6]. In 2014, Helenius-Hietala, *et al*., published a study on oral mucosal lesions in a group of liver transplant recipients. They compared the prevalence of oral mucosal lesions in groups of patients with different etiologies of liver disease using various immunosuppressive agents with a normal control group. The results of this study were in line with our study in terms of oral mucosal lesions such as candidiasis, angular cheilitis, and ulcers [14]. Other studies have also reported high mucosal lesions such as fissured tongue in liver transplant recipients [8]. The fact is that after organ transplantation all patients are placed on high-dose immunosuppressive and corticosteroid medications. These drugs have different mucosal manifestations. Therefore, other manifestations such as gingival overgrowth and lichenoid drug reactions were seen in patients studied in Finland and not in our study group.

Hyposalivation, defined as decreased unstimulated saliva secretion <0.1 g/min [17], was not detected in our patients. However, the mean saliva secretion was significantly lower in patients than the comparison group. The same results are reported by another study where 48% of patients with liver cirrhosis experienced reduced saliva flow rates. However, in that study flow rates <1 mL/min were considered “reduced,” and no control group was evaluated [18]. In addition, 56% of the patients had a reduction in saliva secretion. In that study, 70% of the patients were taking diuretics for the treatment of liver cirrhosis-associated ascites, which was mentioned as the main cause of hyposalivation [6]. In the current study, 11% of patients were using antihypertensive drugs, and 9% tranquilizers, which reduce saliva secretion. Hyposalivation predisposes the patient to various oral and dental disease and infections [14]. Therefore, vigorous oral hygiene instructions should be presented to this group to avoid further problems. 

Regarding dental status, the mean DMFT score was 15.2, which was higher in the patients compared with the comparison group. This was in accordance with other studies that found reduced oral health status in liver transplant candidates [2, 18]. A previous study shows that the mean DMFT score is 22.5 in patients with liver cirrhosis, and that the severity of liver disease augments this score [18]. In 2012, a prospective study compared the severity and etiology of liver disease prior to transplantation with oral health status of patients. They concluded that etiology and severity of disease (MELD score) is related to oral health status in such a way that a worse dental health is seen in those with higher MELD scores, and that patients with primary biliary cirrhosis have the highest tooth loss. They also found that age is associated with the number of tooth extractions [2]. The MELD score has been validated in different patient groups with end-stage liver disease. Because this score predicts the risk of death and the severity of the liver disease, many countries use it for allocation for liver transplantation. We did not find any correlations between the MELD score and oral health status. This difference could be attributed to the larger sample size and longer follow-up of other studies. 

In 2007, Guggenheimer, *et al*. found that dental health characteristics of patients with chronic liver failure were quite similar to those of the normal population. The main risk factor for untreated dental disease was not being visited for dental examination within the past 12 months [6]. The main causes of poor oral health in this group of patients mentioned in literature, are old age, preoccupation with systemic and medical issues, reduced motivation, and depression and inability to fulfill necessary health regimens [6]. In addition, periapical lesions of teeth related to dental infections were detected in 46% of patients with cirrhosis in 2016. Older age and smoking were contributing factors whereas etiology of liver disease and severity (MELD score) had no significant relation with the manifestation of the lesions. The more prevalent the periapical lesions, the more complications of liver disease such as ascites or liver encephalopathy occurred [19]. Furthermore, a study has reported a correlation between oral infections and accelerated progression of the liver disease as measured by the MELD score [20].

Candidates of liver transplantation are faced with a dose-dependent decrease in the proliferative capacity of osteoblasts with increasing bilirubin levels. A correlation is also found between low levels of insulin-like growth factor and reduced bone formation [21, 22]. Almost 64% of our patients had signs of bone loss. This finding was not significantly more prevalent in cirrhotic patients compared to normal individuals. Other studies have also shown that these group of patients are more prone to osteoporotic changes of the jaw bones [23, 24].

In conclusion, we found that mucosal lesions, especially fungal-related lesions, are more prevalent in the oral cavity of patients with chronic liver failure. This finding had no relation with the etiology of liver disease and also with the severity of the disease (MELD score). Hyposalivation is also a complication seen in cirrhotic patients. The use of various medications predisposes these patients to reduced saliva flow rates. These groups of patients are thus more prone to mucosal and dental infections. Vigorous oral hygiene instructions and frequent dental visit must be prescribed for them prior to transplantation.

## References

[1] Lok AS, McMahon BJ (2009). Chronic hepatitis B: update 2009. Hepatology.

[2] Helenius-Hietala J, Meurman JH, Höckerstedt K (2012). Effect of the aetiology and severity of liver disease on oral health and dental treatment prior to transplantation. Transpl Int.

[3] Smith BW, Adams LA (2011). Non-alcoholic fatty liver disease. Critical Rev Clin Lab Sci.

[4] Guggenheimer J, Eghtesad B, Stock DJ (2003). Dental management of the (solid) organ transplant patient. Oral Surg Oral Med Oral Pathol Oral Radiol Endod.

[5] Guggenheimer J, Mayher D, Eghtesad B (2005). A survey of dental care protocols among US organ transplant centers. Clin transplant.

[6] Guggenheimer J, Eghtesad B, Close JM (2007). Dental health status of liver transplant candidates. Liver transpl.

[7] Rustemeyer J, Bremerich A (2007). Necessity of surgical dental foci treatment prior to organ transplantation and heart valve replacement. Clin oral invest.

[8] Diaz-Ortiz M, Micó-Llorens J, Gargallo-Albiol (2005). Dental health in liver transplant patients. Med Oral Patol Oral Cir Bucal.

[9] Douglas LR, Douglass JB, Sieck JO, Smith PJ (1998). Oral management of the patient with end-stage liver disease and the liver transplant patient. Oral Surg Oral Med Oral Pathol Oral Radiol Endod.

[10] Little JW, Rhodus NL (1992). Dental treatment of the liver transplant patient. Oral surg oral med oral pathol.

[11] Olczak-Kowalczyk D, Pawłowska J, Cukrowska B (2008). Local presence of cytomegalovirus and Candida species vs oral lesions in liver and kidney transplant recipients. Ann transplant.

[12] Becker T, Levin L, Shochat T, Einy S (2007). How Much Does the DMFT Index Underestimate the Need for Restorative Care?. J Dent Educ.

[13] Navazesh M (1993). Methods for collecting saliva. Ann N Y Acad Sci.

[14] Helenius-Hietala J, Ruokonen H, Grönroos L (2014). Oral mucosal health in liver transplant recipients and controls. Liver Transplant.

[15] Appleton SS (2000). Candidiasis: pathogenesis, clinical characteristics, and treatment. J Calif Dent Assoc.

[16] Krishnan PA, Kannan R (2013). Comparative study on the microbiological features of angular cheilitis in HIV seropositive and HIV seronegative patients from South India. J Oral Maxillofac Pathol.

[17] Bergdahl M, Bergdahl J (2000). Low unstimulated salivary flow and subjective oral dryness: association with medication, anxiety, depression, and stress. J Dent Res.

[18] Lins L, Bittencourt PL, Evangelista MA (2011). Oral health profile of cirrhotic patients awaiting liver transplantation in the Brazilian Northeast. Transplant proc.

[19] Gronkjaer LL, Holmstrup P, Schou S (2016). Presence and consequence of tooth periapical radiolucency in patients with cirrhosis. Hepat Med.

[20] Åberg F, Helenius-Hietala J, Meurman J, Isoniemi H (2014). Association between dental infections and the clinical course of chronic liver disease. Hepatol Res.

[21] Karoli Y, Karoli R, Fatima J, Manhar M (2016). Study of Hepatic Osteodystrophy in Patients with Chronic Liver Disease. J Clin Diagn Res.

[22] Patel N, Muñoz SJ (2015). Bone disease in cirrhosis. Clin Liver Dis (Hoboken).

[23] Ghapanchi J, Zahed M, Haghnegahdar A (2018). Osteoporosis and Jaw Abnormalities in Panoramic Radiography of Chronic Liver Failure Patients. Biomed Res Int.

[24] Farzin M, Golchin A, Badie A (2017). The effect of two types of denture adhesive on the satisfaction parameters of complete denture wearers. J Dent Biomater.

